# Human Hemorrhagic Fever Causing Arenaviruses: Molecular Mechanisms Contributing to Virus Virulence and Disease Pathogenesis

**DOI:** 10.3390/pathogens4020283

**Published:** 2015-05-21

**Authors:** Junjie Shao, Yuying Liang, Hinh Ly

**Affiliations:** Department of Veterinary and Biomedical Sciences, University of Minnesota, 1988 Fitch Ave., Ste 295, Saint Paul, MN 55108, USA; E-Mails: jshao@umn.edu (J.S.); liangy@umn.edu (Y.L.)

**Keywords:** arenaviruses, Lassa virus, Junin virus, pathogenic mechanisms, immune evasion, virus replication

## Abstract

Arenaviruses include multiple human pathogens ranging from the low-risk lymphocytic choriomeningitis virus (LCMV) to highly virulent hemorrhagic fever (HF) causing viruses such as Lassa (LASV), Junin (JUNV), Machupo (MACV), Lujo (LUJV), Sabia (SABV), Guanarito (GTOV), and Chapare (CHPV), for which there are limited preventative and therapeutic measures. Why some arenaviruses can cause virulent human infections while others cannot, even though they are isolated from the same rodent hosts, is an enigma. Recent studies have revealed several potential pathogenic mechanisms of arenaviruses, including factors that increase viral replication capacity and suppress host innate immunity, which leads to high viremia and generalized immune suppression as the hallmarks of severe and lethal arenaviral HF diseases. This review summarizes current knowledge of the roles of each of the four viral proteins and some known cellular factors in the pathogenesis of arenaviral HF as well as of some human primary cell-culture and animal models that lend themselves to studying arenavirus-induced HF disease pathogenesis. Knowledge gained from these studies can be applied towards the development of novel therapeutics and vaccines against these deadly human pathogens.

## 1. Introduction

Arenaviruses are ambisense RNA viruses that are divided into Old World (OW) and New World (NW) virus groups based on their phylogenetic, serological, and geographical differences. While the prototypic OW arenavirus LCMV with worldwide distribution causes only mild illness in immunocompetent individuals, two other known OW arenaviruses (LASV and LUJV, both found in Africa) can cause severe hemorrhagic fever (HF) in humans. In addition, several of the NW arenaviruses in South America (e.g., JUNV, MACV, SABV, GTOV, and CHPV) can also cause severe hemorrhagic fevers (HF). There are currently limited preventative and therapeutic options for patients infected with these highly pathogenic viruses. Candid #1 is the only vaccine currently available against JUNV infection [1], but is only licensed for the endemic areas in Argentina. Ribavirin, a nucleoside analog, has been used for the treatment of arenavirus infections but has had mixed success and significant toxicity [2].

Besides the aforementioned pathogenic human arenaviruses, there are many other known and emerging arenaviruses that have no known causal roles in humans [3,4], including but not necessarily limited to a new group of snake-borne arenaviruses in the *Reptarenavirus* genus of the *Arenaviridae* family [5]. An important question is why some arenaviruses cause severe disease in humans, while others do not. Recent studies using viral reverse genetics, cell-based assays, animal models, and human genome-wide association analyses have revealed several potential mechanisms of arenaviral pathogenicity. We will summarize current understanding of the roles of the different viral and cellular factors that contribute to the degrees of arenavirus virulence in humans.

## 2. Human Diseases Caused by OW and NW Arenaviruses

LASV is responsible for up to 300,000 infections and 5000–10,000 deaths annually in endemic areas of West Africa [2]. LASV infection, which is often misdiagnosed, can result in a wide range of disease symptoms ranging from non-symptomatic to multi-organ failure and death. Some general symptoms include fever, cough, sore throat, malaise, severe headache, nausea, vomiting, and diarrhea; these can develop into petechial hemorrhage and facial swelling (edema) [6]. More severe symptoms include pleural effusion, thrombocytopenia, leukopenia, sensorineural hearing loss (which occurs in up to one third of patients), encephalopathy, seizures, coma, mucosal bleeding, pulmonary edema, respiratory distress, and shock that culminates in death of the patients [6,7]. The only other known hemorrhagic fever-causing OW arenavirus, LUJV, was identified during an outbreak of the disease in Lusaka (Zambia) and Johannesburg (Republic of South Africa) in 2008 [8]. LUJV-infected patients experienced fever, diarrhea, vomiting, chest pain, sore throat, rash, myalgia, facial and/or cerebral edema, mild bleeding, respiratory distress, elevated liver transaminases, and thrombocytopenia [9].

The OW LCMV virus is found worldwide because its natural host (*Mus musculus*) has a wide distribution. While most acquired LCMV infections are either asymptomatic or mild (e.g., fever, sore throat, cough, headache, muscle aches, malaise, photophobia, nausea, vomiting, thrombocytopenia, and leukopenia) [10,11], LCMV infections have also been shown to cause neurological symptoms, such as aseptic meningitis or meningoencephalitis [10,11]. Patients with meningitis may experience fever, headache, myalgia, stiff neck, malaise, and nausea, while those with meningoencephalitis can present with more severe neurological symptoms such as confusion and motor-sensory abnormalities. While acquired LCMV infection does not pose a serious threat to immune-competent adults, congenital infection with LCMV can be quite serious. Congenital LCMV infection can result in spontaneous abortion and fetal death, or leave infants with permanent visual and/or neurological impairments. While 35% of reported cases of congenital LCMV infection are fatal [12], we do not know the exact incidence of congenital LCMV infections since infants are not commonly tested for the infection and only serious cases have been reported [13]. In recent years, LCMV has been shown to be an important pathogen that can cause severe symptoms in solid organ-transplanted recipients, including but not necessarily limited to encephalopathy, coagulopathy, thrombocytopenia, leukocytosis, and graft dysfunction. Fourteen cases of LCMV infection in transplant recipients have so far been identified, 11 of which have proven fatal [14,15,16]. Another OW arenavirus (Dandenong virus) was isolated from fatal organ-transplant patients [9].

Several South American NW arenaviruses (e.g., JUNV, MACV, GTOV, CHPV, and SABV) can cause HF with high rates of mortality in humans. While only small numbers of human infections with SABV and CHPV are known [17,18], many cases of human disease have been reported for JUNV, MACV, or GTOV [19,20,21,22,23,24,25]. JUNV is the causative agent of Argentine hemorrhagic fever. JUNV-infected patients may show mild symptoms such as fever, myalgia, mild hypotension, conjunctivitis, petechial hemorrhage of the soft tissues, lethargy, and irritability. However, in severe cases, patients can experience hemorrhagic fever, leukopenia, thrombocytopenia, shock, and seizures [24]. Other pathogenic NW arenaviruses (e.g., MACV, SABV, GTOV, and CHPV) can also cause a wide range of symptoms, ranging from fever, sore throat, headache, myalgia, nausea, abdominal pain, vomiting, diarrhea, mucosal, and/or conjuntival petechia to more severe symptoms such as thrombocytopenia, leukopenia, hematuria, tremors, pulmonary edema, respiratory distress, coma, shock, and hepatic and gastrointestinal hemorrhages and necrosis. While hepatitis is common in severe cases of Lassa fever, it is unusual or mild in South American hemorrhagic fevers. Neurologic symptoms, hemorrhaging, leukopenia, and thrombocytopenia are more common in JUNV, GTOV, or MACV infections than in LASV infections [26].

## 3. Basic Biology of Arenaviruses

### 3.1. Genome Structure

**Figure 1:**
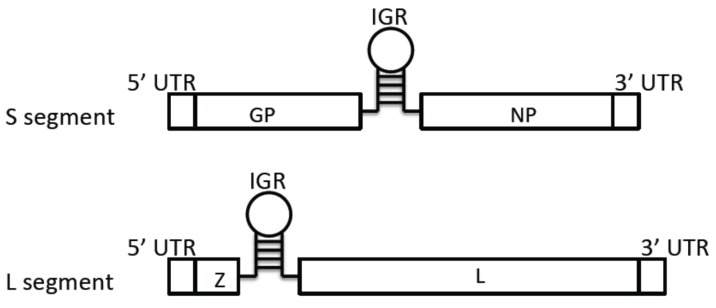
Arenavirus genome structure: Arenaviruses are enveloped RNA viruses with a single-stranded ambisense genome that is composed of two segments. The genomic L (large) segment encodes the Z matrix protein and the L polymerase protein, and the S (small) segment encodes the glycoprotein (GP) and nucleoprotein (NP). The genes encoded on each segment are separated by noncoding intergenic regions (IGR). The linear genomes are flanked by the conserved 5’ and 3’ untranslated regions (UTRs).

**Figure 2:**
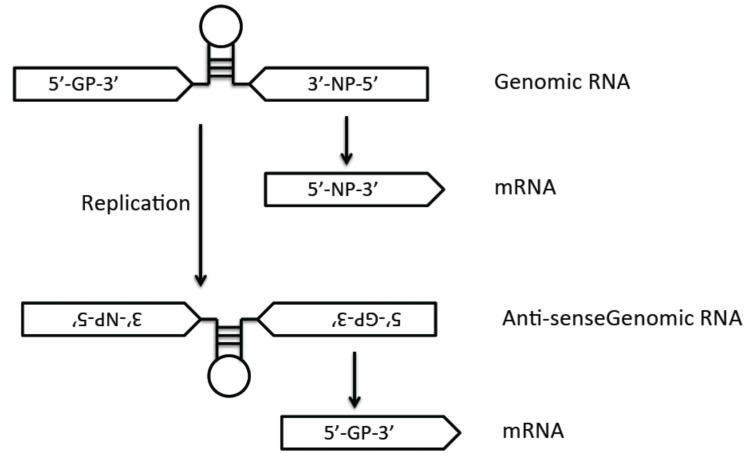
Arenaviral ambisense-genome replication strategy: due to the ambisense coding strategy of the arenaviruses, the NP (and L) genes are transcribed directly from the viral genomic segments into mRNAs, whereas the GP (and Z) mRNAs must be transcribed from the antigenomic strands after genome replication.

The arenavirus genome is composed of two single-stranded negative-sense RNAs of about 7.2 kb and 3.5 kb (Figure 1). Each genomic segment code for the two proteins is from an opposite orientation, which is known as ambisense coding strategy. The large (L) segment encodes the RNA-dependent RNA polymerase (RdRp) L protein and matrix protein Z. The small (S) segment encodes the nucleoprotein (NP) and glycoprotein precursor (GP) [27]. Arenavirus genomic RNA segments do not serve as a direct template for translation. Due to the ambisense coding strategy (Figure 2), the NP and L mRNAs are transcribed from genomic RNAs, whereas the GP and Z viral mRNA are transcribed from the anti-genomic RNAs. The intergenic region (IGR) separates the two genes on each RNA segment, and the hairpin structure of IGR is believed to provide the termination signal of transcription.

The untranslated regions (UTRs) at the 5' and 3' termini of each RNA segment are conserved among arenaviruses. Like other negative-strand RNA viruses, these UTRs are complementary to each other and therefore can form panhandle structures [28]. The UTRs also play an important role in viral nucleocapsid formation and favor the viral polymerase recognition of the viral termini to initiate replication and transcription [28].

### 3.2. Virus Entry Mechanisms

**Figure 3:**
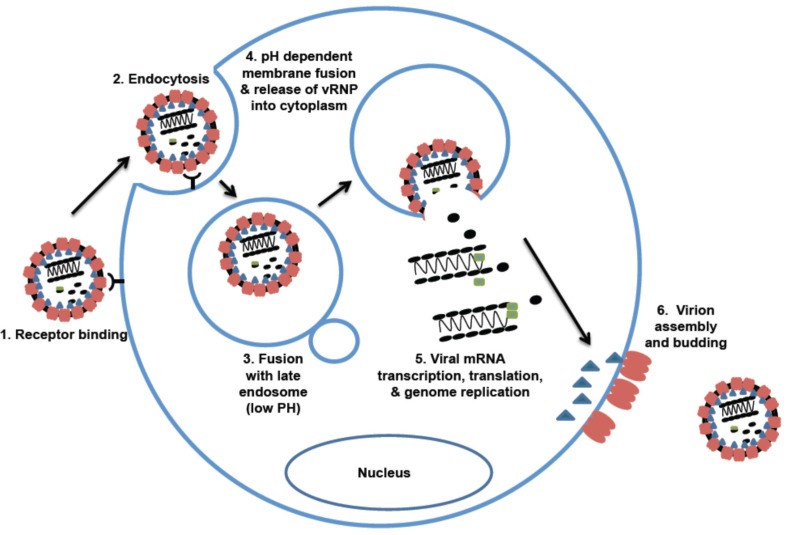
Arenavirus life cycle. 1. Cellular entry is mediated by different cellular receptors (αDG for OW and NW clade C arenaviruses; TfR1 for NW Clade B). Entry of some viruses (e.g., LASV) may also involve a pH-dependent switch to an intracellular receptor (LAMP1) located in the lysosomes. 2. Virus uptake into cells is mediated by endocytosis (OW arenaviruses: clathrin-independent, NW: clathrin-dependent). 3. Virus fusion occurs with the late endosome. 4. Viral RNP is released into the cytoplasm via a pH-dependent membrane fusion mechanism. 5. Viral genome replication, transcription, and protein expression strictly occur in the cytoplasm. 6. Virion assembly and budding occur at the plasma membrane.

The life cycle of arenaviruses starts with virus attachment and entry into cells (Figure 3). The OW and NW clade C arenaviruses use α-dystroglycan (αDG) as their primary receptor [29,30]. Dystroglycan is a ubiquitously expressed glycoprotein that links cells to the extracellular matrix (ECM). It contains two non-covalently associated subunits, αDG and βDG, which play different roles in virus attachment and cellular function. αDG is an extracellular subunit that associates with ECM proteins such as laminin, agrin, perlecan, and neurxins. Upon virus infection, the viral glycoprotein 1 subunit (GP1) mediates attachment to the αDG, which allows the viral particles to internalize and deliver to the late endosomes. βDG is a transmembrane protein that binds to the cytoskeletal adaptor proteins and signaling molecules, but is not required for arenaviral binding and infection [31].

αDG is a highly glycosylated protein, and studies have shown that post-translational modification of αDG is essential for viral binding [31]. Many cellular enzymes are involved in these modification processes, including the cellular like-acetylglucosaminyltransferase (LARGE), LARGE2, putative glycosyltransferases protein O-mannosyltransferase 1/2 (POMT 1/2), protein O-mannose β1,2-N-GlcNAc transferase 1(POMGnT1), fukutin, and fukutin-related protein (FKRP) [32]. An *in vivo* study has shown that glycosylation is not critical for LCMV infection [33]. However, this may be due to the *in vivo* compensation mechanism or the use of an alternate receptor.

LCMV and LASV bind to the N-terminal and C-terminal domains of αDG in regions overlapping the binding site of laminin [34], which suggests that these viruses compete with laminin for αDG binding [35]. Small peptides based on the binding site residues of laminin have been designed and shown to efficiently neutralize these viruses [36]. Interestingly, upon infection by the OW arenaviruses, αDG is downregulated from the cell membrane while the expression of the precursor DG remains unaffected. This is mediated by the viral GP, which targets the interaction between DG and LARGE in the Golgi and thereby disrupts the proper glycosylation of αDG. However, this process may play an important role in viral release and not necessarily at the entry step [32,37].

Animal studies and human clinical data have demonstrated high viral titers in the liver despite the fact that hepatocytes are not known to express αDG [38]. Several cellular factors, including Axl, Tyro3, DC-SIGN, and LSECtin, have been shown to play a role in LASV entry [39]. While a recent study has shown that Axl does not necessarily play a major role in the establishment and maintenance of a persistent LCMV-ARM infection in mice [40], its expression is highly upregulated in mice infected with the LCMV-WE strain, which can induce transient liver pathology [41]. It is also interesting to note that skeletal muscle expresses high levels of αDG without any evidence of virus replication [42]. A recent study has demonstrated that LCMV can replicate in myotubes, but that its entry is restricted in a similar manner to what has been known for avian cells, which also express high levels of αDG but are resistant to arenavirus infection [43]. More recently, the cellular lysosomal-associated membrane protein 1 (LAMP1) has been demonstrated as an intracellular receptor for LASV. It has also been shown that a single glycosylation site of LAMP1 is critical for viral binding, which is absent in birds [44]. Therefore, entry of some viruses (e.g., LASV) may also involve a pH-dependent switch to an intracellular receptor (LAMP1) located in the lysosomes of human and mouse cells [44]. It remains to be determined whether LAMP1 is required for the entry of other arenaviruses.

The NW clade B arenaviruses use transferrin receptor 1 (TfR1) as their receptor [45]. Five viruses in this clade—JUNV, MACV, GTOV, SABV, and CHAPV—can cause HF infections due partly to their ability to bind to the human TfR1 receptor [45,46,47,48]. In contrast, three other viruses in this clade, Tacaribe virus (TCRV), Amapari virus (AMAV), and Cupixi virus (CPXV), are non-pathogenic partly because they cannot bind to hTfR1 but can still use TfR1 orthologs for entry [49]. A recent study has demonstrated that the NW clade A/B arenaviruses use rodent TfR1 as a viral receptor, but that some members of this arenavirus clade may also have the potential to use hTfR1 for entry [50,51,52]. Therefore, hTfR1 receptor usage alone is not the primary determinant of pathogenesis for NW arenaviruses. TfR1 is an iron-transferrin receptor that is regulated by the cytosolic level of iron. Upon transferrin binding to the TfR1, the complex is internalized and delivered to the acidic endosome via the clathrin-mediated pathway. At least three different viral families use TfR1 as host receptors [53,54]. These viruses use a similar strategy to gain the entry into cells [55]. Under low iron dietary condition in some endemic areas, in which TfR1 gene expression can be upregulated, resulting in a suspected worse disease prognosis [56]. Some NW clade B arenaviruses may use alternative receptors for entry. For example, JUNV has been shown to use DC-SIGN and L-SIGN for virus binding and entry [57].

Upon binding to the cellular receptor arenaviruses are internalized by vesicles, and are then released into the cytoplasm through a pH-dependent membrane fusion step that is accomplished by the transmembrane portion of the viral glycoprotein GP2 [58]. The arenaviral glycoprotein subunit 2 (GP2) is a class I fusion protein [59]. However, different from other class I transmembrane proteins, the arenaviral glycoproteins (GPs) remain associated with the unusually long stable signal peptide (SSP) on the membrane. The interaction between SSP and GPs is required for the GP2 membrane-fusion function [60]. The SSP helps keep the GP pre-fusion conformation in the natural environment and facilitates the GP2 response to the acidified environment of the endosome. Interestingly, some studies have shown that LCMV and LASV do not pass through the early endosome. Instead, these OW arenaviruses go directly into the late endosomes, thereby avoiding recognition by the host endosomal immune sensors in the early endosome. As such, infection by the OW arenaviruses, such as LASV, is able to suppress the innate immune response and produce significantly low levels of type I interferons (IFNs) at the early stage of the infection.

Many studies have focused on the process of internalization of the virion particles into the late endosome. By gold labeling LCMV and visualizing it in a transmission electron microscope (TEM), LCMV has been found in smooth and uncoated plasma membranes, close to the primary endocytic vesicles [61]. Few viral particles are found to be associated with clathrin-coated structures. However, a separate study has demonstrated that LCMV infection is independent of clathrin, dynamin-2, actin, Arf6, flotillin-1, caveolae, and lipid rafts. The pathway to deliver LCMV and LASV into acidified endosomes is Rab5-independent but microtubular-dependent [61,62]. This pathway is associated with the cellular endosomal sorting complex required for transport (ESCRT) [62]. This unusual pathway of virus internalization is related to the process of αDG receptor degradation [62].

### 3.3. Viral Genome Replication and Transcription

Arenaviral genome replication and transcription occur in the cytoplasm of the infected cells (Figure 3). Arenaviruses produce three types of RNA species: genomic RNA (gRNA), anti-genomic RNA (agRNA), and viral mRNA. While gRNAs and agRNAs are replicated as full length RNAs, the mRNAs are transcribed from the promoter and terminated by the highly structured IGRs (Figure 2). Because of the ambisense coding strategy, the NP and L mRNAs are directly transcribed from the gRNAs, but the GP and Z mRNAs can only be transcribed from the agRNAs. The NP and L proteins are necessary and sufficient to mediate viral RNA transcription and replication [63,64,65].

The arenaviral L protein contains the viral RdRp, which can be divided into three or four domains based on different types of analysis [66,67,68]. The four conserved RdRp residues are located in the central domain, which mediates viral gene transcription and replication [69]. The C terminal domain of the L protein may be involved in viral mRNA synthesis process, the exact mechanism of which is unknown [70]. The N terminal domain of the L protein has endonuclease activity [71,72], which has been shown to be essential for viral gene transcription but not replication [71]. Arenaviruses employ a “cap snatching” process to obtain the 5' cap structure from cellular mRNAs for use to prime viral mRNA transcription [73]. We and other researchers have recently determined the three-dimensional structures of the endonuclease domain of the LASV and LCMV L proteins [71,72,74]. Both arenaviral NP and L proteins may be involved in this cap-snatching process. The N terminal domain of NP has a deep groove that can potentially bind the m7GpppN cap structure from the cellular mRNAs [74]. This cap binding function of NP is believed to provide some arenaviruses (e.g., JUNV, TCRV, and PICV) with the ability to translate viral genes independently of the cellular eIF4E protein subunit of the eIF4F complex [75]. The NP-NP and NP-L interactions also play an important role in mediating viral mRNA synthesis [76]. Arenaviral matrix Z protein has also been shown to play an important regulatory role in viral RNA replication process [64,77,78,79]. Z can directly interact with the viral L polymerase at the terminal panhandle RNA structure and thereby locks the polymerase in a promoter-bound catalytically inactive state to inhibit viral RNA synthesis [80].

### 3.4. Post-Translational Protein Processing

The nascent viral GP precursor polypeptides are cleaved into the stable signal peptide (SSP) and the G1/G2 peptide in the endoplasmic reticulum (ER) by the cell signal peptidase [60,81]. The GP1/GP2 peptide remains associated with SSP in the ER until it is cleaved further by the cellular protease subtilisin/kexin isozyme-1 (SKI-1)/site 1 protease (S1P) into GP1 and GP2 subunits [81,82]. This process can take place in either the ER or Golgi [82,83,84,85]. Both GP1 and GP2 contain several N-glycosylation sites, which are important for the correct glycoprotein complex (GPC) cleavage, maturation, and transportation. The LASV GPC contains six N-glycosylation sites that are essential for GPC cleavage, but other glycosylation sites do not seem to be directly involved in the proteolytic process [86]. The myristoylation site on the G2 residue of the Z protein is important for the virus budding process due to Z homo-oligomerization [87].

### 3.5. Virion Assembly

Like most enveloped negative-strand RNA viruses, the matrix protein mediates the virion assembly process, which depends in part on its late domain motifs (PPxY, PT/SAP, YxxL, θPxV, where x is any amino acid and θ is a hydrophobic amino acid) [88,89,90]. Sequence analysis shows that most of arenaviral Z proteins contain both the PT/SAP and YxxL motifs with most of the OW arenaviral Z proteins containing the PPxY motif.

The interactions between Z and other viral and cellular proteins are important for the viral assembly and budding process [91]. Several studies have revealed some important domains or residues that are important for these interactions. The myristoylation on the G2 residue is required for the membrane association of Z with GPC [92]. The RING domain and the L79 residue of the TCRV Z protein are required for the interaction between NP and Z [93]. The Z-NP interaction has also been shown for LCMV and LASV [94] and is mediated by the C terminal domain of NP [93,95]. We have confirmed that the G2 myristoylation site, the conserved cysteine and histidine residues of the RING domain, and the L79 and P80 residues in the C terminal domain are essential for Pichinde arenavirus (PICV) replication [96]. The interaction between Z and L has also been demonstrated [80,87] as described above. The homo-oligomerization of Z has been shown to be important for virion budding [97]. Finally, the interactions between the late domain motifs of the Z protein with the cellular ESCRT and/or ESCRT-associated ubiquitin ligase help drive the virus budding process [98]. The LCMV IRG has also been shown to be important for the viral assembly and budding process [99].

## 4. Molecular Mechanisms Contributing to Virus Virulence and HF Disease Pathogenesis

### 4.1. Roles of the Glycoprotein (GP) and of the Host Cell Receptors in HF Disease Susceptibility and Pathogenesis

The ability of a virus to bind to different cellular receptors via its glycoprotein makes GP an important pathogenic factor. OW and NW clade C arenaviruses use alpha-dystroglycan (αDG) as their receptor for cell entry. The differences in affinity of GP binding to αDG may contribute to differences in the disease outcome. Two strains of LCMV, clone 13 (Cl 13) and Armstrong (ARM), can induce different disease outcomes in mouse models. The Cl 13 strain causes chronic infection, whereas the ARM strain causes acute infection. Studies showed that LCMV with high affinity for αDG, such as the Cl 13 strain, is able to infiltrate the white pulp of the spleen and abolish the cytotoxic T lymphocytes, therefore inducing a persistent infection. During the infection of αDG low affinity strain, ARM strain, the viruses are mainly restricted in the red pulp and are quickly cleared by the strong cytotoxic T lymphocyte (CTL) response [34,100].

A recent analysis of over 3 million human genomic polymorphisms by the International HapMap Project has suggested a natural positive selection in a Nigerian population for allele variants of several human genes, including LARGE, interleukin 21 (IL21), and dystrophin (DMD), which is a cytosolic adaptor protein that is required for the proper function of αDG [101,102]. A high proportion of individuals in this population (21%) have shown evidence of prior exposure to LASV, which may apply selective pressure on the allelic frequencies of these human genes, giving rise to their differential gene expressions, which would confer natural resistance to Lassa fever. These polymorphisms in LARGE and DMD, for example, may have hindered binding and entry of LASV, and thereby protected these individuals from severe LASV infection [101,102].

NW clade B arenaviruses include both pathogenic and non-pathogenic viruses. Among this group of viruses, JUNV, MACV, GTOV, SABV, and CHPV can cause severe and lethal HF in humans [17,18,21,25,103,104]. All five of these pathogenic NW clade B arenaviruses use human transferrin receptor 1 (hTfR1) as their receptor for cellular entry [3]. In contrast, the known NW clade B non-pathogenic viruses can only use the rodent version of TfR1 as their receptor. Likewise, the NW clade A/B viruses also use the host-species-specific TfR1 as their receptors [3]. Virion RNAs with sequence homology to the Whitewater Arroyo virus (WWAV), a clade A/B virus, have been detected in several human cases, suggesting that WWAV might be associated with human infections. While some studies have shown that this virus is unable to use hTfR1 as an entry receptor [105,106], WWAV-like viruses have been shown to potentially use hTfR1 as a receptor [3]. However, it is not clear whether usage of hTfR1 plays any role in disease pathogenesis, as WWAV has not directly been shown to be a causative HF agent.

Further evidence for the involvement of GPC as an important determinant of arenavirus virulence has been demonstrated in the guinea pig model for PICV infection [107]. Two strains of PICV (P2 and P18) can cause vastly different disease outcomes in infected animals [108,109]. Mutational analysis of the virulent (P18) and avirulent (P2) strains has revealed that sequence differences between the GPCs may contribute to the different disease outcomes. Substituting a single amino acid residue at position 140 of GPC from the avirulent P2 into the virulent P18 genome can increase the survival rate of infected animals from 0% to 33%, suggesting that GPC plays an important role in the disease outcomes [107]. A separate study has shown that mutational analysis at residue 427 of the GPC of the attenuated Candid #1 strain of JUNV is largely responsible for the attenuation of this vaccine strain in suckling mice [110]. That the residue change at this site results in increased virus-cell fusion at neutral pH of the Candid #1 strain and also in increased dependence on hTfR1 for entry demonstrates its role in contributing to virus attenuation in humans [111]. The study of the ML29 vaccine, a reassortant virus consisting of the LASV S genomic RNA segment and the Mopeia (MOPV) L genomic RNA segment, shows the attenuate phenotype of this reassortant virus in non-human primates and guinea pigs [112,113]. This data suggests that the major virulence factor(s) of LASV are located on the L segment. Other virus reassortant studies using LCMV or PICV have also implicated the contribution of the L genomic RNA segment in viral pathogenesis [114,115,116]. While the L polymerase gene, encoded on the L segment may play an important role, it is important to note that the L segment also encodes the Z protein with known roles in regulating RNA synthesis and host innate immunity that can participate in disease pathogenesis [77,80,117,118]. Interestingly, sequence analysis of the ML29 viruses isolated from infected animals reveals several important mutations on the S segment, especially within the GP coding sequence. The K272E mutation located between the SKI-1/S1P cleavage site and GP2 fusion domain is one such mutation [119]. It remains to be determined whether this and perhaps other mutations within the GP gene or elsewhere on the S segment of the ML29 vaccine virus contribute to the reduced pathogenic phenotype observed for this reassortant virus.

### 4.2. Role of the Polymerase (L) Protein in Viral Virulence

A hallmark of severe and lethal arenavirus-induced HF is the high level of viremia. It has been shown that individuals who can control the level of viremia are able to recover from the infection, while those who cannot typically succumb to the disease [120]. A single amino acid in the L polymerase (L1079) of the LCMV Cl13 has been shown to enhance the levels of intracellular viral replication, which can account for the difference of viral replication rates between the ARM and Cl 13 strains [121]. This residue has also been shown to be responsible for generalized immune suppression, which is likely a result of T cell exhaustion caused by the high viral loads, and therefore may be responsible for the difference in acute *versus* chronic infection by the ARM and Cl 13 strains. A separate study has shown that this same residue is responsible for enhanced viral replication and tropism in macrophages [122]. Using reverse genetics technology, we and other researchers have also demonstrated the role of the L polymerases of LASV and PICV in increasing viral replication and virulence in infected animals [123,124]. We show that the C terminal domain of L polymerase is the virulence determinant of the PICV P18 strain. In particular, three residues (N1906, N1889, and L1839) in the C terminal domain are associated with increased viral replication and pathogenesis in infected animals [124]. It is important to note that all naturally occurring residues found to enhance virus replication in LCMV, LASV, and PICV do not map to the known catalytic domains of the polymerase, and therefore their exact contributions to increased viral RNA synthesis and disease pathogenesis have yet to be determined.

### 4.3. Role of the Nucleoprotein (NP) in Innate Immune Suppression

Host immune responses to infection have a profound and direct influence on the disease course and outcomes. One of the hallmarks of severe and lethal arenavirus-induced HF is generalized immune suppression, the mechanisms of which are still under intense investigation. The nucleoproteins (NPs) of several arenaviruses have been shown to be capable of inhibiting the host innate immune responses. Specifically, the NPs of the OW arenaviruses LCMV and LASV and the NW arenaviruses JUNV, MACV, WWAV, PICV, TCRV, and LATV have been shown to inhibit the production of interferon-beta (IFNβ) [125,126].

Using X-ray crystallography and various functional assays, we and other researchers have demonstrated that the C terminus of the NPs of LASV, TCRV, and PICV contains a functional 3’–5’ exoribonuclease (RNase) domain that degrades dsRNA *in vitro* [74,126,127]. We have therefore proposed that NP inhibits type I IFNs by degrading viral pattern-associated molecular pattern (PAMP) molecules that would otherwise be recognized by cellular pattern recognition receptors (PRRs). The fact that the DEDDH catalytic residues are conserved among all known arenaviruses and that its function to suppress IFNβ production has been demonstrated for both pathogenic (e.g., LASV and LCMV) and non-pathogenic arenaviruses (e.g., TCRV and MOPV) suggests that this is a general mechanism of innate immune suppression by these viruses, allowing them to replicate in either their natural hosts (e.g., rodents) or in infected humans.

Amino acid substitutions of the conserved RNase catalytic residues (DEDDH) significantly reduce NP’s ability to inhibit IFNβ production in virus-infected cell cultures and in animals, directly implicating the exoribonuclease function in innate immune suppression [74,126] We have recently generated recombinant PICVs carrying individual NP RNase catalytic mutations (D380, E382, D457, D525, or H520) [128]. *In vitro* studies of these mutant PICVs show that these viruses induce high levels of IFNβ and grow poorly in the IFN competent A549 cells, but they can grow to similarly high titers as that of the wild-type PICV in IFN-defective Vero cells. All the RNase catalytic mutant viruses show an attenuated phenotype in infected guinea pigs, but show reversion to the wild-type NP sequence in isolates from moribund animals, providing strong genetic evidence for the important role of the NP RNase function in suppressing innate immunity and allowing the virus to replicate in the infected animals. This is consistent with another study in which a recombinant LASV carrying a double-point mutation of the NP RNase catalytic residues (D389 and G392) replicates to lower titers than the wild-type virus partly because it is unable to suppress type I IFNs [129]. The same group of investigators has also recently demonstrated that the LASV NP RNase domain is required to mount an effective antigen-presenting-cell-mediated response in NK cells against virus infection [130]. Other studies have suggested that when NP is overexpressed in cells, they can either prevent activation of the Nuclear Factor Kappa B (NFκB) or directly associate with RIG-I or IKKε in order to inhibit the phosphorylation of the IKKε or of the IFN-responsive gene (IRF3) and its translocation into the nucleus, and thereby negatively regulates type I IFN production [131,132,133]. Taken together, these data confirm the critical role of the arenaviral NP in mediating innate immune suppressive function, which would then allow the virus to replicate unchecked. While the generalized immunosuppression and high viremia are important factors in arenavirus-induced HF pathogenesis, it is important to note that many individuals infected with pathogenic arenaviruses (e.g., LASV) do recover from the infection as a result of a robust cell-mediated immunity [4,6].

### 4.4. Roles of the Z Protein in Innate Immune Suppression and Viral Pathogenicity

In addition to mediating viral budding and regulating viral RNA synthesis (see above), the Z protein also has immune suppressive function. A previous study has shown that the Z proteins of NW arenaviruses (JUNV, MACV, TCRV, and SABV), but not OW arenaviruses LASV and LCMV, can interact with the cytoplasmic pathogen-recognition-receptor RIG-I to inhibit the type I IFN induction [118]. We have recently conducted a comprehensive analysis of the Z proteins from many known arenaviruses and have found that the Z proteins of all nine known pathogenic arenaviruses, including LASV, LCMV, LUJV, JUNV, MACV, TCRV, SABV, and DANV, can effectively suppress the RIG-I-like receptor (RLR)-induced IFN responses [117]. This Z-mediated RLR inhibition is strongly associated with arenavirus pathogenicity, as only the Z proteins of all known pathogenic arenaviruses, but not any of the 14 non-pathogens, can inhibit human RLRs [117]. The inhibition is mediated by the interaction between the flexible N-terminal domain (NTD) of the pathogenic Z proteins and N-terminal tandem CARD domains of RIG-I/MDA5, thus disrupting the association of the RLRs and the mitochondrial antiviral signaling protein MAVS. Swapping of the 31-residue Z NTD from LCMV into a nonpathogenic PICV genome has led to the inhibition of type I IFN responses and increased viral replication in human macrophages—the early target cells of arenavirus infections. This study indicates that the pathogenic Z-mediated RLR inhibition by arenaviruses may be a common pathogenic mechanism underlying the diverse arenavirus family to cause diseases in humans. While each arenavirus pathogen may have its unique pathogenesis leading to variable disease symptoms in humans, all of them encode a Z protein that can inhibit the RLR signaling and thus, the induction of type-I IFNs.

## 5. *In vivo* and *in vitro* Primary Cell-Culture Models of Arenavirus-Induced HF Disease Pathogenesis

### 5.1. Human Primary Cell-Culture Models

Macrophages and dendritic cells (DCs) are known early target cells of arenavirus infection. Several studies have suggested that pathogenic arenaviruses (e.g., LASV and JUNV) and non-pathogenic viruses (e.g., MOPV and TCRV) can differentially inhibit human macrophages but they all can inhibit DCs [117,134,135,136,137,138], the molecular mechanism of which is not yet clearly understood. Both LASV and MOPV have shown to be capable of infecting primary human macrophages and DCs, but intriguingly the pathogenic LASV does not activate these cells upon infection. In sharp contrast, infection of macrophages by the non-pathogenic MOPV activates these cells, as demonstrated by increased expression levels of activation markers CD86, CD80 and IFNα [137]. Similarly, primary human macrophages show higher IL-6, IL-10, and TNFα production upon infection by the nonpathogenic TCRV as compared to the pathogenic JUNV infection [138]. Consistent with these data, our laboratory has recently shown that the Z proteins of all known pathogenic arenaviruses are able to inhibit activation of macrophages, unlike those Z proteins from most known non-pathogenic arenaviruses [117]. Since macrophages and DCs are professional antigen-presenting cells that offer the first line of defense against viral infections, inhibition of these immune cells can induce a general immune suppression of both the innate and adaptive arms of immunity that undoubtedly impact HF disease pathogenesis. Additional studies to investigate the mechanism(s) of immune responses of macrophages and DCs to pathogenic and nonpathogenic arenaviruses are therefore warranted in order to elucidate the interplays between these virus pathogens and host immunity in driving HF disease pathogenesis.

### 5.2. Surrogate and Non-Human Primate Models

Although the pathogenesis of Lassa fever is still not clearly understood, severe LASV infection in humans often results in high levels of viremia, lymphopenia, functional liver damage, vascular abnormalities, and profound suppression of both innate and adaptive immune responses [6]. Different laboratory animal species have been used to mimic key pathophysiological features of human HF diseases [139,140,141]. For example, guinea pigs have been used to study arenavirus-induced lung pathology [142,143], while common marmosets [144] and LCMV-WE-infected rhesus macaques have been used to study arenavirus-induced liver pathology. Due to the strict requirement to work with the highly pathogenic arenaviruses (e.g., LASV) in the high biocontainment (BSL-4) facilities, surrogate animal models of arenaviral HF that involve infection of guinea pigs with the PICV, a risk group 2 pathogen, have been established in outbred Hartley as well as inbred strain 13 guinea pigs to study some mechanisms of Lassa fever-like disease pathogenesis [108,145,146]. When outbred Hartley guinea pigs were infected with a low passage 2 (P2) strain of PICV, it induced a mild disease, whereas a highly adapted P18 strain (passaged 18 strain) of PICV caused a fatal disease with symptoms mimicking some features of Lassa fever, including but not necessarily limited to suppression of proinflammatory cytokines, which was associated with terminal shock and death. While these surrogate animal models can serve as a relatively inexpensive and safe alternative for studying HF disease pathogenesis, the immune system of these rodents can be fundamentally different from those of humans and non-human primates (NHPs). LASV-infected rhesus and cynomolgus monkeys are therefore considered the gold-standard models to study Lassa HF disease pathogenesis HF [38,147,148,149,150,151,152,153,154]. Recent studies focusing on early stages of Lassa fever in NHPs and immune responses [153,154] have confirmed previous observations on several factors important in HF disease pathogenesis, including high viremia, elevated liver enzymes, low or undetectable levels of proinflammatory cytokines (IL-1β, TNF-α, IL-8 and IP-10), and low and/or ineffective T-cell activation. These studies also show that early and strong T-cell responses are associated with effective control of virus replication and recovery. However, much work still needs to be done in order to understand the molecular mechanism(s) underlying HF disease pathogenesis in these animal models.

## 6. Conclusions

Arenaviruses are RNA viruses with a relatively simple genomic structure but with a complex biology and pathogenic mechanisms. The genome of the viruses encodes for only four genes, but each viral protein has multiple functions in mediating optimal viral replication and is possibly involved in the determination of different disease outcomes in humans. Studies have suggested that arenaviruses can gain virulence in hosts by increasing viral cell entry and replication capacity and by effectively suppressing host innate immune responses. The recent identification of the Z protein as a pathogenicity-associated factor has shed important insights into a common pathogenic mechanism underlying the diverse human arenavirus pathogens. Nevertheless, different human arenavirus pathogens exhibit unique features in basic viral replication mechanisms and disease manifestations [4], suggesting that further studies are warranted in order to understand the virus-specific pathogenic mechanisms for individual arenavirus pathogens. Important insights from recent studies on this group of important human pathogens can be exploited for the development of effective preventative and/or therapeutic modalities that can be tested in some established animal models of arenaviral HF.

## References

[1] Maiztegui J.I., McKee K.T., Oro J.G.B., Harrison L.H., Gibbs P.H., Feuillade M.R., Enria D.A., Briggiler A.M., Levis S.C., Ambrosio A.M. (1998). Protective efficacy of a live attenuated vaccine against Argentine hemorrhagic fever. J. Infect. Dis..

[2] Günther S., Lenz O. (2004). Lassa virus. Crit. Rev. Clin. Lab. Sci..

[3] Zong M., Fofana I., Choe H. (2014). Human and host species transferrin receptor 1 use by North American arenaviruses. J. Virol..

[4] McLay L., Liang Y., Ly H. (2014). Comparative analysis of disease pathogenesis and molecular mechanisms of New World and Old World arenavirus infections. J. Gen. Virol..

[5] Radoshitzky S.R., Bao Y., Buchmeier M.J., Charrel R.N., Clawson A.N., Clegg C.S., DeRisi J.L., Emonet S., Gonzalez J.P., Kuhn J.H. (2015). Past, present, and future of arenavirus taxonomy. Arch. Virol..

[6] Moraz M.-L., Kunz S. (2010). Pathogenesis of arenavirus hemorrhagic fevers. Expert Rev. Anti-infective Ther..

[7] Cummins D., McCormick J.B., Bennett D., Samba J.A., Farrar B., Machin S.J., Fisher-Hoch S.P. (1990). Acute sensorineural deafness in Lassa fever. JAMA: J. Am. Med. Assoc..

[8] Briese T., Paweska J.T., McMullan L.K., Hutchison S.K., Street C., Palacios G., Khristova M.L., Weyer J., Swanepoel R., Egholm M. (2009). Genetic detection and characterization of Lujo virus, a new hemorrhagic fever–associated arenavirus from Southern Africa. PLoS Pathogens.

[9] Paweska J.T., Sewlall N.H., Ksiazek T.G., Blumberg L.H., Hale M.J., Lipkin W.I., Weyer J., Nichol S.T., Rollin P.E., McMullan L.K. (2009). Nosocomial outbreak of novel arenavirus infection, Southern Africa. Emerg. Infect. Dis..

[10] Peters C.J. (2006). Lymphocytic choriomeningitis virus — an old enemy up to new tricks. N. Engl. J. Med..

[11] Rousseau M.C., Saron M.F., Brouqui P., Bourgeade A. (1997). Lymphocytic choriomeningitis virus in Southern France: Four case reports and a review of the literature. Eur. J. Epidemiol..

[12] Wright R., Johnson D., Neumann M., Ksiazek T.G., Rollin P., Keech R.V., Bonthius D.J., Hitchon P., Grose C.F., Bell W.E. (1997). Congenital lymphocytic choriomeningitis virus syndrome: A disease that mimics congenital toxoplasmosis or cytomegalovirus infection. Pediatrics.

[13] Bonthius D.J. (2012). Lymphocytic choriomeningitis virus: An underrecognized cause of neurologic disease in the fetus, child, and adult. Semin. Pediatr. Neurol..

[14] Fischer S.A., Graham M.B., Kuehnert M.J., Kotton C.N., Srinivasan A., Marty F.M., Comer J.A., Guarner J., Paddock C.D., DeMeo D.L. (2006). Transmission of lymphocytic choriomeningitis virus by organ transplantation. N. Engl. J. Med..

[15] MacNeil A., Stroher U., Farnon E., Campbell S., Cannon D., Paddock C.D., Drew C.P., Kuehnert M., Knust B., Gruenenfelder R. (2012). Solid organ transplant-associated lymphocytic choriomeningitis, United States, 2011. Emerg. Infect. Dis..

[16] Prevention C.F.D.C.A. (2008). Brief report: Lymphocytic choriomeningitis virus transmitted through solid organ transplantation - Massachussetts, 2008. MMWR Morb Mortal Wkly Rep..

[17] Delgado S., Erickson B.R., Agudo R., Blair P.J., Vallejo E., Albariño C.G., Vargas J., Comer J.A., Rollin P.E., Ksiazek T.G. (2008). Chapare virus, a newly discovered arenavirus isolated from a fatal hemorrhagic fever case in Bolivia. PLoS Pathogens.

[18] Lisieux T., Coimbra M., Nassar E.S., Burattini M.N., Souza L.T.M.D., Ferreira I.B., Rocco I.M., Rosa A.P.A.T.D., Vasconcelos P.F.C., Pinheiro F.P. (1994). New arenavirus isolated in Brazil. Lancet.

[19] Aguilar P.V., Camargo W., Vargas J., Guevara C., Roca Y., Felices V., Laguna-Torres V.A., Tesh R., Ksiazek T.G., Kochel T.J. (2009). Reemergence of Bolivian hemorrhagic fever, 2007–2008. Emerg. Infect. Dis..

[20] Ambrosio A., Saavedra M., Mariani M., Gamboa G., Maiza A. (2011). Argentine hemorrhagic fever vaccines. Hum. Vaccines.

[21] Charrel R.N., Lamballerie X.D. (2003). Arenaviruses other than Lassa virus. Antivir. Res..

[22] Enria D.A., Briggiler A.M., Sánchez Z. (2008). Treatment of Argentine hemorrhagic fever. Antivir. Res..

[23] Fulhorst C.F., Cajimat M.N.B., Milazzo M.L., Paredes H., de Manzione N.M.C., Salas R.A., Rollin P.E., Ksiazek T.G. (2008). Genetic diversity between and within the arenavirus species indigenous to Western Venezuela. Virology.

[24] Harrison L.H., Halsey N.A., McKee K.T., Peters C.J., Barrera Oro J.G., Briggiler A.M., Feuillade M.R., Maiztegui J.I. (1999). Clinical case definitions for Argentine hemorrhagic fever. Clin. Infect. Dis..

[25] Manzione N.D., Salas R.A., Paredes H., Godoy O., Rojas L., Araoz F., Fulhorst C.F., Ksiazek T.G., Mills J.N., Ellis B.A. (1998). Venezuelan hemorrhagic fever: Clinical and epidemiological studies of 165 cases. Clin. Infect. Dis..

[26] Pfau C.J., Baron S. (1996). Arenaviruses. Medical Microbiology.

[27] Meyer B.J., de la Torre J.C., Southern P.J. (2002). Arenaviruses: Genomic RNAs, transcription, and replication. Curr. Top. Microbiol. Immunol..

[28] Knipe D.M.H., Peter M. (2007). Fields Virology.

[29] Cao W., Henry M.D., Borrow P., Yamada H., Elder J.H., Ravkov E.V., Nichol S.T., Compans R.W., Campbell K.P., Oldstone M.B. (1998). Identification of alpha-dystroglycan as a receptor for lymphocytic choriomeningitis virus and Lassa fever virus. Science.

[30] Spiropoulou C.F., Kunz S., Rollin P.E., Campbell K.P., Oldstone M.B. (2002). New world arenavirus Clade C, but not Clade A and B viruses, utilizes alpha-dystroglycan as its major receptor. J. Virol..

[31] Kunz S., Campbell K.P., Oldstone M.B. (2003). Alpha-dystroglycan can mediate arenavirus infection in the absence of beta-dystroglycan. Virology.

[32] Rojek J.M., Spiropoulou C.F., Campbell K.P., Kunz S. (2007). Old World and Clade C New World arenaviruses mimic the molecular mechanism of receptor recognition used by α-dystroglycan's host-derived ligands. J. Virol..

[33] Imperiali M., Sporri R., Hewitt J., Oxenius A. (2008). Post-translational modification of {alpha}-dystroglycan is not critical for lymphocytic choriomeningitis virus receptor function *in vivo*. J. Gen. Virol..

[34] Kunz S., Sevilla N., McGavern D.B., Campbell K.P., Oldstone M.B. (2001). Molecular analysis of the interaction of LCMV with its cellular receptor [alpha]-dystroglycan. J. Cell Biol..

[35] Kunz S., Rojek J.M., Perez M., Spiropoulou C.F., Oldstone M.B. (2005). Characterization of the interaction of Lassa fever virus with its cellular receptor alpha-dystroglycan. J. Virol..

[36] Kunz S., Calder L., Oldstone M.B. (2004). Electron microscopy of an alpha-dystroglycan fragment containing receptor sites for lymphocytic choriomeningitis virus and laminin, and use of the receptoid body as a reagent to neutralize virus. Virology.

[37] Rojek J.M., Campbell K.P., Oldstone M.B.A., Kunz S. (2007). Old World arenavirus infection interferes with the expression of functional α-dystroglycan in the host cell. Mol. Biol. Cell.

[38] Walker D., McCormick J., Johnson K., Webb P., Komba-Kono G., Elliott L., Gardner J. (1982). Pathologic and virologic study of fatal Lassa fever in man. Am. J. Pathol..

[39] Shimojima M., Ströher U., Ebihara H., Feldmann H., Kawaoka Y. (2012). Identification of cell surface molecules involved in dystroglycan-independent Lassa virus cell entry. J. Virol..

[40] Sullivan B.M., Welch M.J., Lemke G., Oldstone M.B. (2013). Is the TAM receptor Axl A receptor for lymphocytic choriomeningitis virus?. J. Virol..

[41] Beier J.I., Jokinen J.D., Holz G.E., Whang P.S., Martin A.M., Warner N.L., Arteel G.E., Lukashevich I.S. (2015). Novel mechanism of arenavirus-induced liver pathology. PloS ONE.

[42] Ibraghimov-Beskrovnaya O., Milatovich A., Ozcelik T., Yang B., Koepnick K., Francke U., Campbell K.P. (1993). Human dystroglycan: Skeletal muscle cDNA, genomic structure, origin of tissue specific isoforms and chromosomal localization. Hum. Mol. Genet..

[43] Lukashevich I.S. (1983). Reproduction of Lassa virus in different cell cultures. Acta Virol..

[44] Jae L.T., Raaben M., Herbert A.S., Kuehne A.I., Wirchnianski A.S., Soh T.K., Stubbs S.H., Janssen H., Damme M., Saftig P. (2014). Virus entry. Lassa virus entry requires a trigger-induced receptor switch. Science.

[45] Radoshitzky S.R., Abraham J., Spiropoulou C.F., Kuhn J.H., Nguyen D., Li W., Nagel J., Schmidt P.J., Nunberg J.H., Andrews N.C. (2007). Transferrin receptor 1 is a cellular receptor for New World haemorrhagic fever arenaviruses. Nature.

[46] Flanagan M.L., Oldenburg J., Reignier T., Holt N., Hamilton G.A., Martin V.K., Cannon P.M. (2008). New World Clade B arenaviruses can use transferrin receptor 1 (TfR1)-dependent and -independent entry pathways, and glycoproteins from human pathogenic strains are associated with the use of TfR1. J. Virol..

[47] Rojek J.M., Kunz S. (2008). Cell entry by human pathogenic arenaviruses. Cell. Microbiol..

[48] Helguera G., Jemielity S., Abraham J., Cordo S.M., Martinez M.G., Rodriguez J.A., Bregni C., Wang J.J., Farzan M., Penichet M.L. (2012). An antibody recognizing the apical domain of human transferrin receptor 1 efficiently inhibits the entry of all New World hemorrhagic fever arenaviruses. J. Virol..

[49] Radoshitzky S.R., Kuhn J.H., Spiropoulou C.F., Albarino C.G., Nguyen D.P., Salazar-Bravo J., Dorfman T., Lee A.S., Wang E., Ross S.R. (2008). Receptor determinants of zoonotic transmission of New World hemorrhagic fever arenaviruses. Proc. Natl. Acad. Sci. USA.

[50] Centers for Disease Control and Prevention (CDC) (2000). Fatal illnesses associated with a New World arenavirus—California, 1999–2000. MMWR Morb Mortal Wkly Rep..

[51] Enserink M. (2000). Emerging diseases. New arenavirus blamed for recent deaths in California. Science.

[52] Milazzo M.L., Campbell G.L., Fulhorst C.F. (2011). Novel arenavirus infection in humans, United States. Emerg. Infect. Dis..

[53] Hueffer K., Parker J.S., Weichert W.S., Geisel R.E., Sgro J.Y., Parrish C.R. (2003). The natural host range shift and subsequent evolution of canine parvovirus resulted from virus-specific binding to the canine transferrin receptor. J. Virol..

[54] Ross S.R., Schofield J.J., Farr C.J., Bucan M. (2002). Mouse transferrin receptor 1 is the cell entry receptor for mouse mammary tumor virus. Proc. Natl. Acad. Sci. USA.

[55] Demogines A., Abraham J., Choe H., Farzan M., Sawyer S.L. (2013). Dual host-virus arms races shape an essential housekeeping protein. PLoS Biol..

[56] Choe H., Jemielity S., Abraham J., Radoshitzky S.R., Farzan M. (2011). Transferrin receptor 1 in the zoonosis and pathogenesis of New World hemorrhagic fever arenaviruses. Curr. Opin. Microbiol..

[57] Martinez M.G., Bialecki M.A., Belouzard S., Cordo S.M., Candurra N.A., Whittaker G.R. (2013). Utilization of human DC-SIGN and l-SIGN for entry and infection of host cells by the New World arenavirus, Junin virus. Biochem. Biophys. Res. Commun..

[58] Eschli B., Quirin K., Wepf A., Weber J., Zinkernagel R., Hengartner H. (2006). Identification of an N-terminal trimeric coiled-coil core within arenavirus glycoprotein 2 permits assignment to class I viral fusion proteins. J. Virol..

[59] Gallaher W.R., DiSimone C., Buchmeier M.J. (2001). The viral transmembrane superfamily: Possible divergence of arenavirus and filovirus glycoproteins from a common RNA virus ancestor. BMC Microbiol..

[60] York J., Nunberg J.H. (2007). Distinct requirements for signal peptidase processing and function in the stable signal peptide subunit of the Junín virus envelope glycoprotein. Virology.

[61] Quirin K., Eschli B., Scheu I., Poort L., Kartenbeck J., Helenius A. (2008). Lymphocytic choriomeningitis virus uses a novel endocytic pathway for infectious entry via late endosomes. Virology.

[62] Pasqual G., Rojek J.M., Masin M., Chatton J.-Y., Kunz S. (2011). Old World arenaviruses enter the host cell via the multivesicular body and depend on the endosomal sorting complex required for transport. PLoS Pathogens.

[63] Hass M., Gölnitz U., Müller S., Becker-Ziaja B., Günther S. (2004). Replicon system for Lassa virus. J. Virol..

[64] Lee K.J., Novella I.S., Teng M.N., Oldstone M.B.A., de la Torre J.C. (2000). NP and L proteins of lymphocytic choriomeningitis virus (LCMV) are sufficient for efficient transcription and replication of LCMV genomic RNA analogs. J. Virol..

[65] López N., Jácamo R., Franze-Fernández M.T. (2001). Transcription and RNA replication of Tacaribe virus genome and antigenome analogs require N and L proteins: Z protein is an inhibitor of these processes. J. Virol..

[66] Vieth S., Torda A.E., Asper M., Schmitz H., Günther S. (2004). Sequence analysis of L RNA of Lassa virus. Virology.

[67] Brunotte L., Lelke M., Hass M., Kleinsteuber K., Becker-Ziaja B., Günther S. (2011). Domain structure of Lassa virus L protein. J. Virol..

[68] Brunotte L., Kerber R., Shang W., Hauer F., Hass M., Gabriel M., Lelke M., Busch C., Stark H., Svergun D.I. (2011). Structure of the Lassa virus nucleoprotein revealed by X-ray crystallography, small-angle X-ray scattering, and electron microscopy. J. Biol. Chem..

[69] Poch O., Sauvaget I., Delarue M., Tordo N. (1989). Identification of four conserved motifs among the RNA-dependent polymerase encoding elements. EMBO J..

[70] Lehmann M., Pahlmann M., Jerome H., Busch C., Lelke M., Gunther S. (2014). Role of the C terminus of Lassa virus L protein in viral mRNA synthesis. J. Virol..

[71] Morin B., Coutard B., Lelke M., Ferron F., Kerber R., Jamal S., Frangeul A., Baronti C., Charrel R., de Lamballerie X. (2010). The N-terminal domain of the arenavirus L protein is an RNA endonuclease essential in mRNA transcription. PLoS Pathogens.

[72] Wallat G.D., Huang Q., Wang W., Dong H., Ly H., Liang Y., Dong C. (2014). High-resolution structure of the N-terminal endonuclease domain of the Lassa virus L polymerase in complex with magnesium ions. PLoS ONE.

[73] Raju R., Raju L., Hacker D., Garcin D., Compans R., Kolakofsky D. (1990). Nontemplated bases at the 5' ends of Tacaribe virus mRNAs. Virology.

[74] Qi X., Lan S., Wang W., Schelde L.M., Dong H., Wallat G.D., Ly H., Liang Y., Dong C. (2010). Cap binding and immune evasion revealed by Lassa nucleoprotein structure. Nature.

[75] Linero F., Welnowska E., Carrasco L., Scolaro L. (2013). Participation of eIF4F complex in Junin virus infection: Blockage of eIF4E does not impair virus replication. Cell. Microbiol..

[76] D'Antuono A., Loureiro M.E., Foscaldi S., Marino-Buslje C., Lopez N. (2014). Differential contributions of Tacaribe arenavirus nucleoprotein N-terminal and C-terminal residues to nucleocapsid functional activity. J. Virol..

[77] Cornu T.I., de la Torre J.C. (2001). Ring finger Z protein of lymphocytic choriomeningitis virus (LCMV) inhibits transcription and RNA replication of an LCMV S-segment minigenome. J. Virol..

[78] Cornu T.I., Feldmann H., de la Torre J.C. (2004). Cells expressing the ring finger Z protein are resistant to arenavirus infection. J. Virol..

[79] Cornu T.I., de la Torre J.C. (2002). Characterization of the arenavirus ring finger Z protein regions required for Z-mediated inhibition of viral RNA synthesis. J. Virol..

[80] Kranzusch P.J., Whelan S.P.J. (2011). Arenavirus Z protein controls viral RNA synthesis by locking a polymerase–promoter complex. Proc. Natl. Acad. Sci. USA.

[81] Eichler R., Lenz O., Strecker T., Garten W. (2003). Signal peptide of Lassa virus glycoprotein GPC exhibits an unusual length. FEBS Lett..

[82] Beyer W.R., Pöpplau D., Garten W., von Laer D., Lenz O. (2003). Endoproteolytic processing of the lymphocytic choriomeningitis virus glycoprotein by the subtilase SKI-1/S1P. J. Virol..

[83] Burri D.J., Pasqual G., Rochat C., Seidah N.G., Pasquato A., Kunz S. (2012). Molecular characterization of the processing of arenavirus envelope glycoprotein precursors by subtilisin kexin isozyme-1/site-1 protease. J. Virol..

[84] Lenz O., ter Meulen J., Klenk H.-D., Seidah N.G., Garten W. (2001). The Lassa virus glycoprotein precursor GPC is proteolytically processed by subtilase SKI-1/S1P. Proc. Natl. Acad. Sci. USA.

[85] Wright K.E., Spiro R.C., Burns J.W., Buchmeier M.J. (1990). Post-translational processing of the glycoproteins of lymphocytic choriomeningitis virus. Virology.

[86] Eichler R., Lenz O., Garten W., Strecker T. (2006). The role of single N-glycans in proteolytic processing and cell surface transport of the Lassa virus glycoprotein GPC. Virol. J..

[87] Loureiro M.E., Wilda M., Levingston Macleod J.M., D'Antuono A., Foscaldi S., Buslje C.M., Lopez N. (2011). Molecular determinants of arenavirus Z protein homo-oligomerization and l polymerase binding. J. Virol..

[88] Bieniasz P.D. (2006). Late budding domains and host proteins in enveloped virus release. Virology.

[89] Im Y.J., Kuo L., Ren X., Burgos P.V., Zhao X.Z., Liu F., Burke T.R., Bonifacino J.S., Freed E.O., Hurley J.H. (2010). Crystallographic and functional analysis of the ESCRT-I/HIV-1 gag PTAP interaction. Structure.

[90] Freed E.O. (2002). Viral late domains. J. Virol..

[91] Loureiro M.E., D’Antuono A., Levingston Macleod J.M., López N. (2012). Uncovering viral protein-protein interactions and their role in arenavirus life cycle. Viruses.

[92] Capul A.A., Perez M., Burke E., Kunz S., Buchmeier M.J., de la Torre J.C. (2007). Arenavirus Z-glycoprotein association requires Z myristoylation but not functional RING or late domains. J. Virol..

[93] Casabona J.C., Levingston Macleod J.M., Loureiro M.E., Gomez G.A., Lopez N. (2009). The RING domain and the L79 residue of Z protein are involved in both the rescue of nucleocapsids and the incorporation of glycoproteins into infectious chimeric arenavirus-like particles. J. Virol..

[94] Ortiz-Riaño E., Cheng B.Y.H., de la Torre J.C., Martínez-Sobrido L. (2011). The C-terminal region of lymphocytic choriomeningitis virus nucleoprotein contains distinct and segregable functional domains involved in NP-Z interaction and counteraction of the type I interferon response. J. Virol..

[95] Levingston Macleod J.M., D'Antuono A., Loureiro M.E., Casabona J.C., Gomez G.A., Lopez N. (2011). Identification of two functional domains within the arenavirus nucleoprotein. J. Virol..

[96] Wang J., Danzy S., Kumar N., Ly H., Liang Y. (2012). Biological roles and functional mechanisms of arenavirus Z protein in viral replication. J. Virol..

[97] Kentsis A., Gordon R.E., Borden K.L.B. (2002). Self-assembly properties of a model RING domain. Proc. Natl. Acad. Sci. USA.

[98] Wolff S., Ebihara H., Groseth A. (2013). Arenavirus budding: A common pathway with mechanistic differences. Viruses.

[99] Pinschewer D.D., Perez M., de la Torre J.C. (2005). Dual role of the lymphocytic choriomeningitis virus intergenic region in transcription termination and virus propagation. J. Virol..

[100] Smelt S.C., Borrow P., Kunz S., Cao W., Tishon A., Lewicki H., Campbell K.P., Oldstone M.B.A. (2001). Differences in affinity of binding of lymphocytic choriomeningitis virus strains to the cellular receptor α-dystroglycan correlate with viral tropism and disease kinetics. J. Virol..

[101] Andersen K.G., Shylakhter I., Tabrizi S., Grossman S.R., Happi C.T., Sabeti P.C. (2012). Genome-wide scans provide evidence for positive selection of genes implicated in Lassa fever. Philos. Trans. R. Soc. B: Biol. Sci..

[102] Sabeti P.C., Varilly P., Fry B., Lohmueller J., Hostetter E., Cotsapas C., Xie X., Byrne E.H., McCarroll S.A., Gaudet R. (2007). Genome-wide detection and characterization of positive selection in human populations. Nature.

[103] Peters C.J. (2002). Human infection with arenaviruses in the Americas. Curr. Top. Microbiol. Immunol..

[104] Maiztegui J.I. (1975). Clinical and epidemiological patterns of Argentine haemorrhagic fever. Bull World Health Organ.

[105] Abraham J., Kwong J.A., Albariño C.G., Lu J.G., Radoshitzky S.R., Salazar-Bravo J., Farzan M., Spiropoulou C.F., Choe H. (2009). Host-species transferrin receptor 1 orthologs are cellular receptors for nonpathogenic New World Clade B arenaviruses. PLoS Pathogens.

[106] Reignier T., Oldenburg J., Flanagan M.L., Hamilton G.A., Martin V.K., Cannon P.M. (2008). Receptor use by the Whitewater Arroyo virus glycoprotein. Virology.

[107] Kumar N., Wang J., Lan S., Danzy S., McLay Schelde L., Seladi-Schulman J., Ly H., Liang Y. (2012). Characterization of virulence-associated determinants in the envelope glycoprotein of Pichinde virus. Virology.

[108] Aronson J., Herzog N., Jerrells T. (1994). Pathological and virological features of arenavirus disease in guinea pigs. Comparison of two Pichinde virus strains. Am. J. Pathol..

[109] Jahrling P.B., Hesse R.A., Rhoderick J.B., Elwell M.A., Moe J.B. (1981). Pathogenesis of a Pichinde virus strain adapted to produce lethal infections in guinea pigs. Infect. Immun..

[110] Albariño C.G., Bird B.H., Chakrabarti A.K., Dodd K.A., Flint M., Bergeron É., White D.M., Nichol S.T. (2011). The major determinant of attenuation in mice of the Candid1 vaccine for Argentine hemorrhagic fever is located in the G2 glycoprotein transmembrane domain. J. Virol..

[111] Droniou-Bonzom M.E., Reignier T., Oldenburg J.E., Cox A.U., Exline C.M., Rathbun J.Y., Cannon P.M. (2011). Substitutions in the glycoprotein (GP) of the Candid#1 vaccine strain of Junin virus increase dependence on human transferrin receptor 1 for entry and destabilize the metastable conformation of GP. J. Virol..

[112] Lukashevich I.S., Carrion R., Salvato M.S., Mansfield K., Brasky K., Zapata J., Cairo C., Goicochea M., Hoosien G.E., Ticer A. (2008). Safety, immunogenicity, and efficacy of the ML29 reassortant vaccine for Lassa fever in small non-human primates. Vaccine.

[113] Carrion R., Patterson J.L., Johnson C., Gonzales M., Moreira C.R., Ticer A., Brasky K., Hubbard G.B., Moshkoff D., Zapata J. (2007). A ML29 reassortant virus protects guinea pigs against a distantly related Nigerian strain of Lassa virus and can provide sterilizing immunity. Vaccine.

[114] Harnish D.G., Dimock K., Bishop D.H., Rawls W.E. (1983). Gene mapping in Pichinde virus: Assignment of viral polypeptides to genomic L and S RNAs. J. Virol..

[115] Riviere Y., Oldstone M.B. (1986). Genetic reassortants of lymphocytic choriomeningitis virus: Unexpected disease and mechanism of pathogenesis. J. Virol..

[116] Lukashevich I.S., Carrion R., Salvato M.S., Mansfield K., Brasky K., Zapata J., Cairo C., Goicochea M., Hoosien G.E., Ticer A. (2008). Safety, immunogenicity, and efficacy of the ML29 reassortant vaccine for Lassa fever in small non-human primates. Vaccine.

[117] Xing J., Ly H., Liang Y. (2015). The Z proteins of pathogenic but not nonpathogenic arenaviruses inhibit RIG-i-like receptor-dependent interferon production. J. Virol..

[118] Fan L., Briese T., Lipkin W.I. (2010). Z proteins of New World arenaviruses bind RIG-i and interfere with type I interferon induction. J. Virol..

[119] Moshkoff D.A., Salvato M.S., Lukashevich I.S. (2007). Molecular characterization of a reassortant virus derived from Lassa and Mopeia viruses. Virus Genes.

[120] Johnson K.M., McCormick J.B., Webb P.A., Smith E.S., Elliott L.H., King I.J. (1987). Clinical virology of Lassa fever in hospitalized patients. J. Infect. Dis..

[121] Bergthaler A., Flatz L., Hegazy A.N., Johnson S., Horvath E., Löhning M., Pinschewer D.D. (2010). Viral replicative capacity is the primary determinant of lymphocytic choriomeningitis virus persistence and immunosuppression. Proc. Natl. Acad. Sci. USA.

[122] Matloubian M., Kolhekar S.R., Somasundaram T., Ahmed R. (1993). Molecular determinants of macrophage tropism and viral persistence: Importance of single amino acid changes in the polymerase and glycoprotein of lymphocytic choriomeningitis virus. J. Virol..

[123] Albariño C.G., Bird B.H., Chakrabarti A.K., Dodd K.A., Erickson B.R., Nichol S.T. (2011). Efficient rescue of recombinant Lassa virus reveals the influence of S segment noncoding regions on virus replication and virulence. J. Virol..

[124] McLay L., Lan S., Ansari A., Liang Y., Ly H. (2013). Identification of virulence determinants within the L genomic segment of the Pichinde arenavirus. J. Virol..

[125] Martínez-Sobrido L., Zúñiga E.I., Rosario D., García-Sastre A., de la Torre J.C. (2006). Inhibition of the type I interferon response by the nucleoprotein of the prototypic arenavirus lymphocytic choriomeningitis virus. J. Virol..

[126] Jiang X., Huang Q., Wang W., Dong H., Ly H., Liang Y., Dong C. (2013). Structures of arenaviral nucleoproteins with triphosphate dsRNA reveal a unique mechanism of immune suppression. J. Biol. Chem..

[127] Hastie K.M., Kimberlin C.R., Zandonatti M.A., MacRae I.J., Saphire E.O. (2011). Structure of the Lassa virus nucleoprotein reveals a dsRNA-specific 3′ to 5′ exonuclease activity essential for immune suppression. Proc. Natl. Acad. Sci. USA.

[128] Huang Q., Shao J., Lan S., Zhou Y., Xing J., Dong C., Liang Y., Ly H. (2015). *In vitro* and *in vivo* characterizations of the Pichinde viral NP exoribonuclease function. J. Virol..

[129] Carnec X., Baize S., Reynard S., Diancourt L., Caro V., Tordo N., Bouloy M. (2011). Lassa virus nucleoprotein mutants generated by reverse genetics induce a robust type I interferon response in human dendritic cells and macrophages. J. Virol..

[130] Russier M., Reynard S., Carnec X., Baize S. (2014). The exonuclease domain of Lassa virus nucleoprotein is involved in antigen-presenting-cell-mediated NK cell responses. J. Virol..

[131] Pythoud C., Rodrigo W.W.S.I., Pasqual G., Rothenberger S., Martínez-Sobrido L., de la Torre J.C., Kunz S. (2012). Arenavirus nucleoprotein targets interferon regulatory factor-activating kinase IKKε. J. Virol..

[132] Rodrigo W.W.S.I., Ortiz-Riaño E., Pythoud C., Kunz S., de la Torre J.C., Martínez-Sobrido L. (2012). Arenavirus nucleoproteins prevent activation of Nuclear factor Kappa b. J. Virol..

[133] Zhou S., Cerny A.M., Zacharia A., Fitzgerald K.A., Kurt-Jones E.A., Finberg R.W. (2010). Induction and inhibition of type I interferon responses by distinct components of lymphocytic choriomeningitis virus. J. Virology.

[134] Mahanty S., Hutchinson K., Agarwal S., Mcrae M., Rollin P.E., Pulendran B. (2003). Cutting edge: Impairment of dendritic cells and adaptive immunity by Ebola and Lassa viruses. J. Immunol..

[135] Baize S., Kaplon J., Faure C., Pannetier D., Georges-Courbot M.-C., Deubel V. (2004). Lassa virus infection of human dendritic cells and macrophages is productive but fails to activate cells. J. Immunol..

[136] Lukashevich I.S., Maryankova R., Vladyko A.S., Nashkevich N., Koleda S., Djavani M., Horejsh D., Voitenok N.N., Salvato M.S. (1999). Lassa and Mopeia virus replication in human monocytes/macrophages and in endothelial cells: Different effects on IL-8 and TNF-alpha gene expression. J. Med. Virol..

[137] Pannetier D., Faure C., Georges-Courbot M.-C., Deubel V., Baize S. (2004). Human macrophages, but not dendritic cells, are activated and produce alpha/beta interferons in response to Mopeia virus infection. J. Virology.

[138] Groseth A., Hoenen T., Weber M., Wolff S., Herwig A., Kaufmann A., Becker S. (2011). Tacaribe virus but not Junin virus infection induces cytokine release from primary human monocytes and macrophages. PLoS Negl. Trop. Dis..

[139] Lukashevich I.S. (2013). The search for animal models for Lassa fever vaccine development. Exp. Rev. Vaccines.

[140] Vela E. (2012). Animal models, prophylaxis, and therapeutics for arenavirus infections. Viruses.

[141] Safronetz D., Geisbert T.W., Feldmann H. (2013). Animal models for highly pathogenic emerging viruses. Curr. Opin. Virol..

[142] Jahrling P.B., Smith S., Hesse R.A., Rhoderick J.B. (1982). Pathogenesis of Lassa virus infection in guinea pigs. Infect. Immun..

[143] Peters C.J., Jahrling P.B., Liu C.T., Kenyon R.H., McKee K.T., Barrera Oro J.G. (1987). Experimental studies of arenaviral hemorrhagic fevers. Curr. Top. Microbiol. Immunol..

[144] Carrion R., Brasky K., Mansfield K., Johnson C., Gonzales M., Ticer A., Lukashevich I., Tardif S., Patterson J. (2007). Lassa virus infection in experimentally infected marmosets: Liver pathology and immunophenotypic alterations in target tissues. J. Virol..

[145] Scott E.P., Aronson J.F. (2008). Cytokine patterns in a comparative model of arenavirus haemorrhagic fever in guinea pigs. J. Gen. Virol..

[146] Liang Y., Lan S., Ly H. (2009). Molecular determinants of Pichinde virus infection of guinea pigs—A small animal model system for arenaviral hemorrhagic fevers. Ann. N. Y. Acad. Sci..

[147] Jahrling P.B., Hesse R.A., Eddy G.A., Johnson K.M., Callis R.T., Stephen E.L. (1980). Lassa virus infection of rhesus monkeys: Pathogenesis and treatment with ribavirin. J. Infect. Dis..

[148] Lange J.V., Mitchell S.W., McCormick J.B., Walker D.H., Evatt B.L., Ramsey R.R. (1985). Kinetic study of platelets and fibrinogen in Lassa virus-infected monkeys and early pathologic events in Mopeia virus-infected monkeys. Am. J. Trop. Med. Hyg..

[149] Callis R.T., Jahrling P.B., DePaoli A. (1982). Pathology of Lassa virus infection in the rhesus monkey. Am. J. Trop. Med. Hyg..

[150] Fisher-Hoch S.P., Mitchell S.W., Sasso D.R., Lange J.V., Ramsey R., McCormick J.B. (1987). Physiological and immunologic disturbances associated with shock in a primate model of Lassa fever. J. Infect. Dis..

[151] Hensley L.E., Smith M.A., Geisbert J.B., Fritz E.A., Daddario-DiCaprio K.M., Larsen T., Geisbert T.W. (2011). Pathogenesis of Lassa fever in cynomolgus macaques. Virol. J..

[152] Safronetz D., Strong J.E., Feldmann F., Haddock E., Sogoba N., Brining D., Geisbert T.W., Scott D.P., Feldmann H. (2013). A recently isolated Lassa virus from Mali demonstrates atypical clinical disease manifestations and decreased virulence in cynomolgus macaques. J. Infect. Dis..

[153] Rasmussen A.L., Tchitchek N., Safronetz D., Carter V.S., Williams C.M., Haddock E., Korth M.J., Feldmann H., Katze M.G. (2015). Delayed inflammatory and cell death responses are associated with reduced pathogenicity in Lujo virus-infected cynomolgus macaques. J. Virol..

[154] Baize S., Marianneau P., Loth P., Reynard S., Journeaux A., Chevallier M., Tordo N., Deubel V., Contamin H. (2009). Early and strong immune responses are associated with control of viral replication and recovery in Lassa virus-infected cynomolgus monkeys. J. Virol..

